# Concomitant Thyroid Disorders and Inflammatory Bowel Disease: A Literature Review

**DOI:** 10.1155/2016/5187061

**Published:** 2016-03-03

**Authors:** Toru Shizuma

**Affiliations:** Department of Physiology, School of Medicine, Tokai University, 143 Shimokasuya, Isehara, Kanagawa 259-1193, Japan

## Abstract

The aim of this report was to review and summarize the literature on cases of concomitant inflammatory bowel disease (IBD) and thyroid diseases. We included the following previous case reports of concomitant IBD and thyroid diseases: 16 cases of ulcerative colitis (UC) and Graves' disease (GD), 3 cases of Crohn's disease (CD) and GD, 10 cases of CD and Hashimoto's thyroiditis (HT), 4 cases of IBD and subacute thyroiditis (SAT) or SAT-like symptoms, and 13 cases of IBD (12/13 cases were CD) and amyloid goiter. There might be no obvious differences of prevalence of thyroid dysfunction (hyper- or hypothyroidism), GD, and thyroid cancer between IBD patients and general populations. However, concomitant UC and HT might be relatively common in patients with multiple autoimmune disorders, and AG is one of the complications with CD patients. There might be no obvious differences of fatal prognoses between IBD patients with thyroid diseases and patients with thyroid diseases without IBD.

## 1. Introduction

Ulcerative colitis (UC) and Crohn's disease (CD) are the two most common inflammatory bowel diseases (IBD). Both UC and CD are chronic recurrent conditions and are characterized by intestinal inflammation that may result from a combination of causes such as environmental or immunological factors [1]. CD can affect any part of the gastrointestinal tract, whereas UC is characterized by inflammation confined to the large intestine [1]. Microscopically, CD affects the entire bowel wall, whereas UC is restricted to the epithelial lining of the gut [1]. Although the development of extraintestinal manifestations or coexistence of autoimmune disorders during the course of IBD is well known, the coexistence of IBD and thyroid diseases has not been well documented.

Thyroid diseases include autoimmune thyroid diseases (ATDs), malignancy, amyloid goiter (AG), subacute thyroiditis (SAT), and congenital thyroid diseases. Thyroid dysfunctions are generally classified into hyperthyroidism or thyrotoxicosis and hypothyroidism. The most common causes of hyperthyroidism are Graves' disease (GD or Basedow disease), excessive supplementation of thyroid hormones, toxic adenoma, and toxic multinodular goiter; nonthyroid diseases may also cause hyperthyroidism [2, 3]. On the other hand, the common causes of hypothyroidism are Hashimoto's thyroiditis (HT or autoimmune thyroiditis), iodine-deficient conditions, postoperative states such as thyroidectomy, and isotope treatment; however, thyroid hormone levels in HT patients often display normal levels [4].

To date, there have been few systematic literature reviews of cases of concomitant thyroid diseases and IBD. Here we conducted a literature search and review to evaluate cases of concomitant IBD and thyroid diseases.

## 2. Methods

We performed a review of the English and Japanese literature regarding the coexistence of thyroid diseases and IBD. We used PubMed and Japana Centra Revuo Medicina (Igaku Chuo Zasshi) for the English and Japanese literature, respectively.

According to the evaluation of thyroid dysfunction, hyperthyroidism is diagnosed when both the serum-free triiodothyronine (fT3) and free thyroxin (fT4) levels are elevated, but the thyroid stimulating hormone (TSH) levels are restrained. Hypothyroidism is diagnosed when serum levels of both fT3 and fT4 are decreased, but the level of TSH is elevated. Moreover, subclinical hyperthyroidism is diagnosed when serum levels of both fT3 and fT4 are within the normal range, but the level of TSH is decreased. Furthermore, subclinical hypothyroidism is diagnosed when serum levels of both fT3 and fT4 are within the normal range, but the level of TSH is elevated.

## 3. Prevalence of Concomitant Thyroid Dysfunction in IBD

### 3.1. Prevalence of Thyroid Dysfunction in General Populations

The studies identified by the literature search indicated a 2%–8% prevalence of thyroid dysfunction (hyper- or hypothyroidism) in the general population, including the populations in iodine-deficient countries [5]. The prevalence of hyperthyroidism in women is between 0.5% and 2% and it is 10 times less common in men [6]. The incidence and prevalence of overt hypothyroidism have been reported to be 2–4/1,000 population/year and 0.8%–1.3%, respectively [7].

### 3.2. Prevalence of Concomitant Thyroid Dysfunction in UC

Some studies have reported a similar prevalence of thyroid dysfunction in UC patients (2.2%–8.0%) compared with general populations [5, 8–12]. Moreover, Casella et al. [5] found that the prevalence of thyroid dysfunction in the general population in Italy was 7.5% (429/5721), which was significantly higher than that in UC patients (2.5%; 4/162). On the other hand, some population studies have demonstrated a two- to fourfold increase in the prevalence of thyroid dysfunction in patients with UC compared with the prevalence in the general population [13–16].

The reported prevalence of hyperthyroidism in UC patients was 0.62%–3.7% [3, 5, 15, 17–21]. On the other hand, the prevalence of UC in patients with hyperthyroidism was 1.34% [22]. Casella et al. [5] also reported that the prevalence of hyperthyroidism in the general population in Italy was 1.21% (69/5721) and was 0.62% (1/162) in UC patients. Snook et al. [15] reported that the prevalence of hyper- and hypothyroidism in UC patients was 1.5% and 0.9%, respectively; however, the prevalence of hyper- and hypothyroidism in the control group was 0.7% for both [15]. Based on the findings of their study, the development of autoimmune disorders, including thyroid diseases, did not show a clear temporal relationship with the onset or activity of IBD, with the exception of concomitant cases of autoimmune hemolytic anemia in UC patients [15]. In addition, a study by Järnerot et al. [16] reported that the prevalence of hyperthyroidism in UC patients was significantly higher compared with that in controls (3.7% versus 0.8%; *p* < 0.01). Recently, there have been few studies that have specifically investigated cases of concomitant hyperthyroidism and UC. These studies are summarized in Tables 1 and 2.

### 3.3. Prevalence of Concomitant Thyroid Dysfunction in CD

Some studies reported that there were no significant differences in thyroid function test (serum levels of fT3, fT4, and TSH) between CD patients and controls [24, 51]. In a study by Snook et al. [15], the prevalence of hyper- and hypothyroidism in CD patients was 0.3% and 0.5%, respectively. In the same study, the prevalence of hyper- and hypothyroidism in the control group was 0.7% for both [15]. Yakut et al. [24] reported that the prevalence of both hyper- and hypothyroidism in patients with CD as well as for both control groups was 0% (0/33) and 0% (0/66), respectively. Liu et al. [19] reported that the prevalence of hyper- and hypothyroidism in patients with CD was 0% (0/44) and 2.3% (1/44), respectively. In a study by Pooran et al. [23], the prevalence of hypothyroidism was lower in CD patients [3.8% (8/210)] than in control individuals [8.2% (17/206)], although the prevalence of hyperthyroidism was statistically similar between the groups. These studies are summarized in Tables 1 and 2.

## 4. Concomitant GD in IBD

### 4.1. GD

GD, also known as Basedow's disease in Europe, is the most common cause of hyperthyroidism [3, 29]. It is one of the most common autoimmune disorders, with an annual incidence of approximately 14 individuals per 100,000 [52]. GD is caused by circulating antibodies (anti-TSH receptor autoantibodies) that mimic the actions of TSH, resulting in the increased synthesis and release of thyroid hormones [52]. GD is associated with extrathyroidal manifestations, including orbital disease (ophthalmopathy), skin changes, and sometimes fingertip and nail abnormalities [53]. The diagnostic criteria for GD include clinical and/or biochemical evidence of thyrotoxicosis and one or more of the following features: (1) the presence of serum TSH receptor autoantibodies (TRAbs), (2) ophthalmopathy and/or dermopathy, and (3) diffuse elevated thyroid radioiodine uptake [52].

### 4.2. Concomitant GD and UC

In general, the pathophysiology of UC is associated with the Th2 cytokine phenotype, and there is increased Th2 activity in GD [21]. Therefore, both GD and UC are associated with a Th1/Th2 imbalance, with a dominance of Th2 responses [13, 21, 22, 28, 29]. Matsumura et al. [21] reported on a 26-year-old female who had a flare-up of UC and hyperthyroidism that was successfully treated with infliximab. In addition, they reported that the Th1/Th2 imbalance was improved 2 weeks after the initiation of infliximab therapy. However, it is still unclear whether GD is an extraintestinal manifestation of UC or not [17]. In addition, some studies have found no differences in the prevalence of hyperthyroidism between UC patients and the general population [5]. Therefore, it is currently unclear whether concomitant GD and UC occur by chance or reflect a common immunological basis [8].

One accepted hypothesis for the pathogenesis of IBD is that the mucosal immune system exhibits an aberrant response toward luminal antigens such as commensal bacteria [54]. For example, a chronic low-grade portal infection, caused by UC and leading to chronic biliary tract inflammation and fibrosis, has been suggested as a pathogenic mechanism [55]. However, because the colon and thyroid do not have the same embryological origins, the same trigger antibodies may not be the cause of the association between GD and UC [13, 21].

According to Casella et al. [5], the first case of concomitant hyperthyroidism and UC was reported in 1968. In total, we identified 16 reported cases of concomitant GD and UC (eight in the English-language literature [5, 13, 14, 17, 18, 27, 30] and eight in the Japanese-language literature [8–11, 25, 26, 28, 29]). The characteristics of these 16 reported cases of concomitant GD and UC are summarized in Table 3. We excluded the cases of concomitant UC and hyperthyroidism that were caused by excessive supplementation of thyroxine after a subtotal thyroidectomy for toxic adenoma, by toxic multinodular goiter, or by unknown causes [56]. Some reports discussed cases of UC induced or aggravated by the administration of rituximab used to treat GD. Therefore, we also excluded the suspected rituximab-induced UC cases with GD [57, 58] from these concomitant UC and GD cases.

Hyperthyroidism is more common in females than in males, with a female-to-male ratio of 10 : 1 [6, 29]. In contrast, UC is not a gender-specific disease; the female-to-male ratio for UC ranges from 0.51 to 1.58 [59]. Of the 16 cases of concomitant GD and UC that were identified in this review, 6 (37.5%) were male and 10 (62.5%) were female. In most cases of concomitant GD and UC that had been reported in the 1980s and 1990s, GD was diagnosed prior to the development of UC. However, in the cases reported in the 2000s, there was no clear tendency in the order of diagnosis. In fact, of the 16 cases reported since 1980, UC developed before GD in six cases (37.5%). In nine cases (56.3%), GD developed before UC, and both UC and CD were simultaneously diagnosed in one case (6.3%). The diagnosis of the concomitant disease occurred between the ages of 18/19 and 61 years, and the time interval between the diagnosis of the primary and concomitant disease was 0–20 years. The types of UC in the 16 cases of concomitant GD and UC included 6 cases of pancolitis and 3 cases of left-sided colitis; there were no cases of proctitis. The type of UC was unclear in seven cases (Table 3).

There were no reports of a flare-up of UC soon after the onset of GD. In most cases of concomitant GD and UC, UC was treated with medications such as aminosalicylates and corticosteroids. Only three of these cases required surgery (colectomy) for persistent colitis despite pharmacotherapy [5, 14, 29]. There were no reports of severe complications of UC such as toxic megacolon in these cases. In most cases, the GD was treated with antithyroid agents and only one case required surgery (subtotal thyroidectomy) [17]. There were no reports of death related to concomitant GD and UC and no evidence that patients with concomitant GD and UC had a poorer prognosis than those with UC but not GD.

### 4.3. Concomitant GD and CD

#### 4.3.1. Genetic Associations between GD and CD

Some studies have assessed possible common genetic factors between GD and CD. The role of non-HLA genes such as PTPN22, CTLA4, and CD40 in GD patients has been extensively investigated [60]. Although some studies have reported that PTPN22 did not influence the risk of IBD, including CD [61], other studies have reported that PTPN22 may influence the risk of developing CD [62, 63]. Moreover, some studies have also reported that CTLA4 may influence the risk of developing CD [64, 65]. In a Spanish meta-analysis, the frequency of the minor allele rs1883832T on the CD40 gene was significantly higher in CD patients than in control individuals, but it was not significantly higher in UC patients [66]. However, further investigations may be necessary to identify any common genetic factors responsible for CD and GD. At present, it is uncertain whether cases of concomitant CD and GD occur due to common genetic backgrounds.

#### 4.3.2. Characteristics of Cases of Concomitant GD and CD

The characteristics of the 3 reported cases of concomitant CD and GD are summarized in Table 4 [22, 31, 32]. Of the three cases of concomitant CD and GD that were identified in this review, two cases were male and one was female. In two cases, CD was diagnosed before the development of GD. The diagnosis of the concomitant diseases was made between the ages of 14 and 38 years, and the interval between the diagnoses of the primary and concomitant diseases was 0–16 years. Only 1 case required surgery (ileotomy) for perforation of the ileum, which had occurred 22 years before the development of GD [22] (Table 4). In all three cases, thyroid dysfunctions were normal after pharmacotherapy. There were no deaths due to CD or GD.

On the other hand, Kettaneh et al. reported on two cases of hyperthyroidism that were caused by adenomatous goiter or Plummer's disease in CD and Takayasu arteritis [56].

## 5. HT in IBD

### 5.1. HT

HT, which is also known as autoimmune thyroiditis, is one of the most common autoimmune endocrine diseases, characterized by an autoimmune-mediated destruction of the thyroid gland and predominantly affecting women [67]. HT may also be characterized by an enlarged thyroid gland and is identified histologically by lymphocytic thyroid infiltration and positive antibody tests for antithyroglobulin and/or antithyroid peroxidase antibodies [4, 67]. HT is a common cause of hypothyroidism. However, some cases exhibit normal thyroid hormone (including TSH) levels, whereas other cases exhibit subclinical hypothyroidism in the presence of increased TSH levels. HT can also cause hyperthyroidism due to destructive (painless) thyroiditis [67]. The diagnosis of HT relies on the demonstration of circulating antibodies to thyroid antigens (mainly thyroperoxidase and thyroglobulin) and reduced echogenicity on the sonography of patients with proper clinical features. The treatment is based on the administration of synthetic thyroid hormones to correct for hypothyroidism [67].

### 5.2. Prevalence of Concomitant HT in IBD

In Japan, the prevalence of HT (chronic thyroiditis) in UC patients was reported as 0.14% (8/5833) during the 1980s and as 0.07% (1/1433) during the 1990s [8, 11]. Bardella et al. [68] reported that the prevalence of HT in UC patients was 2.2% (2/90) and that of HT in CD patients was 4.4% (4/90). Yakut et al. [24] reported that the prevalence of HT in UC patients was 3.5% (4/113) and that HT in CD patients was 0% (0/33). Cesarini et al. [20] also reported that the prevalence of HT in UC patients was 1.8% (8) and that HT in CD patients was 2.2% (10/464) in Italy. In a large population-based study in Canada that included 8072 IBD patients (3879 UC and 4193 CD patients), the prevalence of HT (autoimmune thyroiditis) was similar to that of the controls [69].

### 5.3. Concomitant Cases of HT and UC

Although sporadic cases of concomitant HT and UC have been reported, most reports were cases of autoimmune diseases such as immune thrombocytopenic purpura, type 1 diabetes mellitus, or autoimmune hepatitis other than HT that had also occurred in patients with UC and HT [70, 71]. Moreover, ATDs are one of the components in the multiple autoimmune syndrome, which consists of three or more well-defined autoimmune conditions in the same patient [72]. Moreover, some authors have reported a case with concomitant UC and HT in a patient with Turner syndrome [73, 74]. In a cohort study of 2,459 patients, the risk of concomitant HT and IBD (both UC and CD) was significantly higher in Turner syndrome patients compared with the general population [75]. This suggests that HT and UC can be a manifestation of Turner syndrome.

### 5.4. Concomitant Cases of HT and CD

The characteristics of the 10 previously reported cases of concomitant CD and HT are summarized in Table 5 (eight in the English-language literature [22, 33, 34, 36, 38, 39] and two in the Japanese-language literature [35, 37]). Of these, three cases occurred in males and seven occurred in females. After excluding three cases that were simultaneously diagnosed with CD and HT, CD was diagnosed before the development of HT in five of the remaining seven cases. The concomitant disease was diagnosed between the ages of 10 and 55 years, and the interval between the diagnoses of the primary and concomitant disease was 0–27 years. Regarding thyroid function, there were four cases of hypothyroidism [33, 35, 37], four cases of hyperthyroidism due to suspected destructive (or painless) thyroiditis [22, 36, 38, 39], and one case with normal function [33]; the thyroid function was unclear in one case because the results of thyroid function test (fT3/fT4 and TSH levels) were not mentioned in that paper [34]. The treatment included administration of synthetic thyroid hormones in cases with hypothyroidism. Moreover, no thyroid dysfunction cases resistant to treatments were observed. There were no deaths due to CD or HT. One of the 10 cases represented familial CD [39].

Among the reported cases, Noto et al. [35] described a case of a young female who was diagnosed almost simultaneously with CD and HT; she also had Turner syndrome. As previously discussed, the risk of concomitant HT and IBD (both UC and CD) was significantly higher in Turner syndrome patients compared with the general population [75].

## 6. SAT in IBD

SAT is an uncommon condition, yet it is considered as the most common cause of painful thyroiditis. The disease is believed to have a viral origin, although the precise etiology of SAT is unknown. Common laboratory findings include transient thyrotoxic conditions and poor or no thyroid uptake. Within a few weeks and after the depletion of any preformed thyroid hormone, approximately 30% of patients will undergo a hypothyroid phase [76]. Standard treatment for SAT is administration of prednisolone. Moreover, SAT-like syndrome (symptoms) may have similar clinical symptoms as SAT and the thyroid function in SAT-like syndrome is within the normal range [41, 50, 77]. The development of SAT in IBD patients appears to be extremely rare. A few cases of SAT or SAT-like syndrome have occurred in IBD patients [50, 77, 78]. One case report of SAT in IBD was reported by Horai et al. [78]. They reported on a female UC patient who was diagnosed with SAT and Takayasu's arteritis >20 years after the diagnosis of UC. This suggests that genetic factors may be associated with the occurrence of these conditions [78]. In another case, Kawashima et al. [50] reported a middle-aged man with AG and CD, who developed hyperthyroidism caused by SAT after the administration of the tumor necrosis factor- (TNF-) *α* inhibitor infliximab, and subsequent SAT-like symptoms then developed after the administration of another TNF-*α* inhibitor, adalimumab (suspected drug-induced thyroiditis). However, after an examination of 36 rheumatic patients, Kaklamanos et al. [79] reported that infliximab and rituximab did not cause any alterations in thyroid function and/or autoimmunity (thyroid antibody titer), even in patients with previously undiagnosed autoimmune thyroid diseases. Ikenoue et al. [77] described recurrent SAT-like symptoms in two cases of IBD (one with CD and the other with suspected indeterminate colitis) that were complicated by thyroid amyloidosis. Therefore, SAT or SAT-like syndrome should be considered in IBD patients that have complications such as amyloid deposition in the thyroid. Moreover, in these cases, SAT or SAT-like syndromes resolved after prednisolone treatment.

## 7. AG in IBD

### 7.1. Amyloidosis in IBD

Amyloidosis is characterized by the deposition of fibrillary proteins that can accumulate in various organs [49] and is classified as primary or secondary. Secondary amyloidosis is a well-known complication of CD, as demonstrated by previous studies, and has an incidence of 0.5%–9.0% [42, 48]. However, the prevalence of amyloidosis is less in UC patients. Greenstein et al. [40] reported on 16 cases (0.52%; 15 of CD and 1 of UC) among 3,050 IBD cases and found that the prevalence of amyloidosis was 0.88% (15/1709) in CD patients and was 0.07% (1/1341) in UC patients. They also found that only one case (0.03% of all IBD patients and 0.06% of all CD patients) had AG. Moreover, they reviewed 25 IBD cases (22 of CD and 3 of UC) that were complicated with amyloidosis and reported that renal disturbances occurred in 84% (21/25) of IBD patients; most deaths in this population were associated with renal complications such as nephrotic syndrome or renal failure [40].

### 7.2. AG in IBD

AG, characterized by thyroid enlargement caused by extensive amyloid deposition in the thyroid gland [40, 49, 80], is an uncommon complication of IBD. In most cases, AG is associated with amyloid deposition in other organs during the course of systemic amyloidosis and with the glandular deposition of amyloid AA [81]. AG is clinically characterized by the rapid growth of the thyroid gland, resulting in the development of pressure symptoms [80]. Although thyroid function in AG patients is usually within the normal range, hyper- and hypothyroidism develop sometime [42, 49, 80, 81]. However, Kimura et al. [82] reported that 9 of 10 (90%) AG patients had abnormal thyroid function, including the low T3 syndrome; they also found that five patients had hypothyroidism, one had hyperthyroidism, one had transient hypothyroidism, and two had low T3 syndrome.

### 7.3. AG in UC

Although sporadic cases of CD complicated with AG have been reported, the development of AG in UC patients appears to be extremely rare. To the best of our knowledge, there have been no reports of such concomitant cases in the English literature. However, one such case was discussed in a Japanese proceeding that involved a 35-year-old female with UC and renal amyloidosis (chronic renal failure on hemodialysis) who had developed AG with a slight tendency of hypothyroidism [83]. However, AG did not result in the flare-up of UC.

### 7.4. AG in CD

Our literature review identified 12 reports (seven in English-language [40, 42, 47–50], one in Spanish [46], and four in Japanese-language [41, 43–45]) of CD complicated with AG, which are summarized in Table 6. The cases of CD with focal amyloid deposition and without goiter were excluded from our review. Among these cases, seven occurred in males and four in females; the sex of one patient was unclear [47]. In these cases, AG was diagnosed between the ages of 26 and 58 years. The major clinical symptoms were the development of a painful neck mass and associated swelling. With regard to thyroid function in the 11 cases (excluding the one case in which the data was not available [40]), the thyroid function in eight was within the normal range [41–43, 46–50], two were diagnosed with hypothyroidism [45, 48], and one was diagnosed with subclinical hypothyroidism [44]. These AG cases were diagnosed by histopathological findings and/or Congo red staining after thyroidectomy or fine-needle aspiration biopsy. Among these 12 cases, one case report suggested that the development of goiter was associated with the activity of CD [41]. However, there were no obvious correlations between the development of AG and activity or flare-up of IBD; four cases showed no correlations between the development of goiter and activity or flare-up of CD [42, 44, 48, 50] and these correlations were not mentioned in the remaining seven cases. Standard treatment including diagnosis for AG in concomitant CD and AG cases was surgery (thyroidectomy).

Among these 12 cases, three were complicated with chronic renal failure due to renal amyloidosis [40, 43, 44] and two others were complicated with nephrotic syndrome due to renal amyloidosis [46, 48]. Outcomes of AG in CD patients may be partially dependent on other organ disturbances by amyloid deposition, such as renal failure. Only one death occurred due to renal failure associated with renal amyloidosis [40].

## 8. Thyroid Cancer in IBD

Thyroid cancer includes papillary thyroid cancer (PTC), follicular cancer, medullary cancer, and poorly or undifferentiated differentiated cancer. The case reports of concomitant cases of thyroid cancer [84–89] or primary thyroid lymphoma [90, 91] and IBD have been sporadic. There have been case reports of PTC in patients with UC [85, 86, 88] and CD [84, 87, 89]. Although primary lymphoma of the thyroid is quite rare, its prevalence is increasing in a proportion of cases with HT. Further, Triantafillidis [90] reported a case with concomitant primary thyroid lymphoma and HT in a patient with UC.

Moss et al. [84] reported five cases of PTC in CD patients. Among these cases, one patient had been on immunomodulator therapy before the diagnosis of thyroid cancer. They suspected that exposure to radiation through imaging (computed tomography or small bowel radiological investigations) in early adulthood or while receiving multivitamins might have been the cause [84]. However, none of the patients had a history of thyroid dysfunction, radiation exposure, or family history of thyroid cancer [84]. Radiation exposure at a young age has been a consistent risk factor for PTC, and the risk of PTC is higher in women with a high multivitamin supplement intake [84, 92].

Yano et al. [93] demonstrated that the incidence of thyroid cancer in 770 Japanese CD patients was not significantly higher compared with healthy populations. Moreover, they found that previous 11 studies demonstrated that the incidence of thyroid cancer in CD patients was not significantly higher than that in healthy populations [93]. Sonu et al. [94] suggested that the age at diagnosis of PTC in CD patients was significantly lower than that in UC patients or control populations and that patients with CD and UC were not less likely to develop PTC compared to those control populations in the United States.

## 9. Nonthyroidal Illness Syndrome in IBD

Nonthyroidal illness syndrome (NTIS) is characterized as a significantly low serum level of fT3 in patients with nonthyroidal diseases, such as various acute and chronic diseases [19]. Although there were few studies on NTIS in IBD patients, Liu et al. [19] reported that the prevalence of NTIS in CD patients was relatively common [36.4% (16/44)], and CD patients with NTIS displayed worse nutritional status and clinical outcome. Moreover, compared with CD patients with euthyroidism, these patients also exhibited enhanced critical disease activity and severity.

## 10. Ectopic Thyroid Gland in IBD

An ectopic thyroid gland is a rare congenital abnormality; this condition is caused by the failure of the gland anlage to descend early in the course of embryogenesis. Research has estimated its frequency to be 1/4000 to 1/8000 among patients with hypothyroidism and to be 0.3% among all thyroid diseases [95]. To the best of our knowledge, there have been no cases of concomitant ectopic thyroid gland and IBD.

## 11. Conclusions

In this report, we conducted a literature search and review to evaluate the cases of concomitant IBD and thyroid diseases or thyroid dysfunction, ATDs (GD and HT), SAT, AG, and thyroid cancer. At present, there might be no obvious differences in the prevalence of thyroid dysfunction, GD, and thyroid cancer between IBD patients and the general population. However, concomitant UC and HT might be relatively common in particular patients with multiple autoimmune disorders. Moreover, there might be no correlation between development of thyroid disease and severity of IBD. There might be no obvious differences of fatal prognoses between patients with concomitant thyroid diseases and IBD and patients with thyroid diseases without IBD. However, it will be necessary to gather additional data to clarify these conditions.

## Figures and Tables

**Table 1:** The reported prevalence of hyperthyroidism in inflammatory bowel disease.

Authors (year)	Type of IBD	Prevalence in IBD	Prevalence in general populations	Reference
Järnerot et al. (1975)	UC	3.7% (11/300)^*∗*^	0.83% (5/600)^*∗*^	[16]
Snook et al. (1989)	UC	1.5% (13/858)	0.67% (2/300)	[15]
	CD	0.26% (1/378)	0.67% (2/300)	[15]
Pooran et al. (2003)	CD	0.95% (2/210)	0% (0/206)	[23]
Casella et al. (2008)	UC	0.62% (1/162)	1.2% (69/5721)	[5]
Yakut et al. (2011)	UC	4.4% (5/113)	0% (0/66)	[24]
	CD	0% (0/33)	0% (0/66)	[24]
Liu et al. (2013)	CD	0% (0/44)	—	[19]

IBD: inflammatory bowel disease; UC: ulcerative colitis; CD: Crohn's disease.

^*∗*^The prevalence was significantly higher in UC patients than in the general populations (*p* < 0.01).

**Table 2:** The reported prevalence of hypothyroidism in inflammatory bowel disease.

Authors (year)	Type of IBD	Prevalence in IBD	Prevalence in general populations	Reference
Snook et al. (1989)	UC	0.93% (8/858)	0.67% (2/300)	[15]
	CD	0.53% (2/378)	0.67% (2/300)	[15]
Pooran et al. (2003)	CD	3.8% (8/210)^*∗*^	8.3% (17/206)^*∗*^	[23]
Casella et al. (2008)	UC	1.9% (3/162)^*∗*^	6.3% (360/5721)^*∗*^	[5]
Yakut et al. (2011)	CD	0% (0/33)	0% (0/66)	[24]
Liu et al. (2013)	CD	2.3% (1/44)	—	[19]

IBD: inflammatory bowel disease; UC: ulcerative colitis; CD: Crohn's disease.

^*∗*^The prevalence was significantly higher in the general populations than in IBD patients.

**Table 3:** Characteristics of the patients with concomitant ulcerative colitis and Graves' disease.

Case	(Year)	Gender	Age at diagnosis of UC (years)	Age at diagnosis of GD (years)	UC prior to GD	UC type	Complications	Reference
1	(1980)	F	46 or 47	46	−	Left-sided colitis?		[18]
2	(1980)	M	18 or 19	18	−	Pancolitis?		[18]
3	(1981)^*∗*^	F	53	53 or 54	+	?	Dermatomyositis	[25]
4	(1984)^*∗*^	M	46	36	−	Pancolitis		[9]
5	(1985)^*∗*^	F	66	46	−	Pancolitis		[26]
6	(1996)^*∗*^	F	31?	30	−	Left-sided colitis		[10]
7	(1998)	M	17	24	+	?	Primary sclerosing cholangitis	[27]
8	(1999)^*∗*^	F	32	30	−	?		[11]
9	(2001)	F	41	41	Sim	?		[13]
10	(2001)	M	14	26	+	Pancolitis?		[14]
11	(2001)^*∗*^	F	35	31?	−	Left-sided colitis	Familial GD	[28]
12	(2001)^*∗*^	M	24	26	+	Pancolitis		[8]
13	(2005)	F	42	47	+	?		[17]
14	(2008)	F	61	60	−	?		[5]
15	(2009)^*∗*^	M	22	26	+	Pancolitis	Familial UC	[29]
16	(2012)	F	38	18	−	?	IgA nephropathy	[30]

UC: ulcerative colitis; GD: Graves' disease; F: female; M: male; Sim: simultaneous.

^*∗*^Japanese-language literature.

**Table 4:** Characteristics of the patients with concomitant Crohn's disease and Graves' disease.

Case	(Year)	Gender	Age at diagnosis of CD (years)	Age at diagnosis of GD (years)	CD prior to GD	Thyroid function tests			Remarks	Reference
						TSH (*μ*U/mL)	freeT3 (pg/mL)	freeT4 (ng/dL)		
1	(1999)	M	14	14	Sim	<0.1	?	?		[31]
							→ normalization			
2	(2004)^*∗*^	M	19 or 20	20	+	0.05	5.6	3.7		[32]
							→ normalization			
3	(2005)	F	22	38	+	0.017	>20	>12	Familial GD	[22]
							→ normalization		CD → ileotomy	

CD: Crohn's disease; GD: Graves' disease; F: female; M: male; Sim: simultaneous.

^*∗*^Japanese-language literature.

**Table 5:** Characteristics of the patients with concomitant Crohn's disease and Hashimoto's thyroiditis.

Case	(Year)	Gender	Age at diagnosis of CD (years)	Age at diagnosis of HT (years)	CD prior to HT	Thyroid function tests (TSH: *μ*U/mL, freeT3: pg/mL, freeT4: ng/dL)	Complications	Reference
1	(1988)	M	17	44	+	Hypothyroidism (TSH 5.3) → normalization		[33]
2	(1988)	F	26	43	+	Normal		[33]
3	(1988)	F	43	55	+	Hypothyroidism (TSH 9.6)		[33]
4	(2002)	F	53	46	−	?	Sjögren's syndrome	[34]
5	(2005)	F	16	24	+	Thyrotoxicosis (TSH 0.036, freeT3 13.2, freeT4 5.6) → normalization		[22]
6	(2006)^*∗*^	F	26	26	Sim	Hypothyroidism (TSH 3.90, freeT4 1.39) → normalization	Turner syndrome	[35]
7	(2008)	F	15	15	Sim	Thyrotoxicosis (TSH < 0.05, free T4 2.7) → subsequent hypothyroidism	Beta-thalassemia	[36]
8	(2012)^*∗*^	F	14	10	−	Hypothyroidism → normalization		[37]
9	(2012)	M	10	10	Sim	Thyrotoxicosis (TSH 0.05, freeT4 31.5)	Bardet-Biedl syndrome Primary sclerosing cholangitis	[38]
10	(2013)	M	33?	35?	+	Thyrotoxicosis?	Primary biliary cirrhosis Familial CD	[39]

CD: Crohn's disease; HT: Hashimoto's thyroiditis; F: female; M: male; Sim: simultaneous.

^*∗*^Japanese-language literature.

**Table 6:** Characteristics of amyloid goiter in patients with Crohn's disease.

Case	(Year)	Gender	Age at diagnosis of CD (years)	Age at diagnosis of AG (years)	Thyroid function	Complications	Reference
1	(1992)	F	13	33?	?	Chronic renal failure	[40]
2	(1995)^*∗*^	M	25	26	Within the normal range	Subacute thyroiditis-like symptoms	[41]
3	(1999)	M	15	26	Within the normal range		[42]
4	(2000)^*∗*^	M	16	27	Within the normal range	Chronic renal failure	[43]
5	(2007)^*∗*^	M	?	38	Subclinical hypothyroidism	Chronic renal failure	[44]
6	(2008)^*∗*^	F	25	30<	Hypothyroidism		[45]
7	(2009)	F	40?	47	Within the normal range	Nephrotic syndrome	[46]
8	(2010)	?	?	?	Within the normal range		[47]
9	(2012)	M	<21	44	Within the normal range		[48]
10	(2012)	F	34	46	Slight hypothyroidism	Nephrotic syndrome	[48]
11	(2014)	M	58?	58	Within the normal range		[49]
12	(2015)	M	50	56	Within the normal range	Subacute thyroiditis and subsequent subacute thyroiditis-like symptoms	[50]

CD: Crohn's disease; AG: amyloid goiter; F: female; M: male.

^*∗*^Japanese-language literature.
